# Exploring the retinal connectome

**Published:** 2011-02-03

**Authors:** James R. Anderson, Bryan W. Jones, Carl B. Watt, Margaret V. Shaw, Jia-Hui Yang, David DeMill, James S. Lauritzen, Yanhua Lin, Kevin D. Rapp, David Mastronarde, Pavel Koshevoy, Bradley Grimm, Tolga Tasdizen, Ross Whitaker, Robert E. Marc

**Affiliations:** 1 Department of Ophthalmology, Moran Eye Center, University of Utah, Salt Lake City, UT; 2 The Boulder Laboratory For 3-D Electron Microscopy of Cells, University of Colorado, Boulder, CO; 3 Scientific Computing and Imaging Institute, University of Utah, Salt Lake City, UT; 4 Sorenson Media, Salt Lake City, UT; 5 Department Electrical and Computer Engineering, University of Utah, Salt Lake City, UT

## Abstract

**Purpose:**

A connectome is a comprehensive description of synaptic connectivity for a neural domain. Our goal was to produce a connectome data set for the inner plexiform layer of the mammalian retina. This paper describes our first retinal connectome, validates the method, and provides key initial findings.

**Methods:**

We acquired and assembled a 16.5 terabyte connectome data set RC1 for the rabbit retina at ≈2 nm resolution using automated transmission electron microscope imaging, automated mosaicking, and automated volume registration. RC1 represents a column of tissue 0.25 mm in diameter, spanning the inner nuclear, inner plexiform, and ganglion cell layers. To enhance ultrastructural tracing, we included molecular markers for 4-aminobutyrate (GABA), glutamate, glycine, taurine, glutamine, and the in vivo activity marker, 1-amino-4-guanidobutane. This enabled us to distinguish GABAergic and glycinergic amacrine cells; to identify ON bipolar cells coupled to glycinergic cells; and to discriminate different kinds of bipolar, amacrine, and ganglion cells based on their molecular signatures and activity. The data set was explored and annotated with Viking, our multiuser navigation tool. Annotations were exported to additional applications to render cells, visualize network graphs, and query the database.

**Results:**

Exploration of RC1 showed that the 2 nm resolution readily recapitulated well known connections and revealed several new features of retinal organization: (1) The well known AII amacrine cell pathway displayed more complexity than previously reported, with no less than 17 distinct signaling modes, including ribbon synapse inputs from OFF bipolar cells, wide-field ON cone bipolar cells and rod bipolar cells, and extensive input from cone-pathway amacrine cells. (2) The axons of most cone bipolar cells formed a distinct signal integration compartment, with ON cone bipolar cell axonal synapses targeting diverse cell types. Both ON and OFF bipolar cells receive axonal veto synapses. (3) Chains of conventional synapses were very common, with intercalated glycinergic-GABAergic chains and very long chains associated with starburst amacrine cells. Glycinergic amacrine cells clearly play a major role in ON-OFF crossover inhibition. (4) Molecular and excitation mapping clearly segregates ultrastructurally defined bipolar cell groups into different response clusters. (5) Finally, low-resolution electron or optical imaging cannot reliably map synaptic connections by process geometry, as adjacency without synaptic contact is abundant in the retina. Only direct visualization of synapses and gap junctions suffices.

**Conclusions:**

Connectome assembly and analysis using conventional transmission electron microscopy is now practical for network discovery. Our surveys of volume RC1 demonstrate that previously studied systems such as the AII amacrine cell network involve more network motifs than previously known. The AII network, primarily considered a scotopic pathway, clearly derives ribbon synapse input from photopic ON and OFF cone bipolar cell networks and extensive photopic GABAergic amacrine cell inputs. Further, bipolar cells show extensive inputs and outputs along their axons, similar to multistratified nonmammalian bipolar cells. Physiologic evidence of significant ON-OFF channel crossover is strongly supported by our anatomic data, showing alternating glycine-to-GABA paths. Long chains of amacrine cell networks likely arise from homocellular GABAergic synapses between starburst amacrine cells. Deeper analysis of RC1 offers the opportunity for more complete descriptions of specific networks.

## Introduction

Connectomics has the potential to be a Rosetta Stone for neuroscience in that it may decode the wiring of any brain region [1,2]. We recently described a framework for automated transmission electron microscope (ATEM) imaging of large-scale neural assemblies [3] and tools for connectome data mining [4]. Here, we here report the assembly, initial analysis, and open-access availability of RC1, which is the first practical connectome data set from the mammalian retina. To be useful, ultrastructural connectomics requires a near-canonical sample of processing elements [3], cell classification with high coverage [5], and resolution sufficient to track all connections. The size of such data sets [3,6,7], in turn, requires high-speed acquisition. All of these needs are met by ATEM imaging. In particular, RC1 contains a large sample of the rabbit retinal inner plexiform layer (IPL), which includes molecular markers of cell identity and activity, and has sufficient resolution to identify all synapses and most gap junctions.

**Figure 1:**
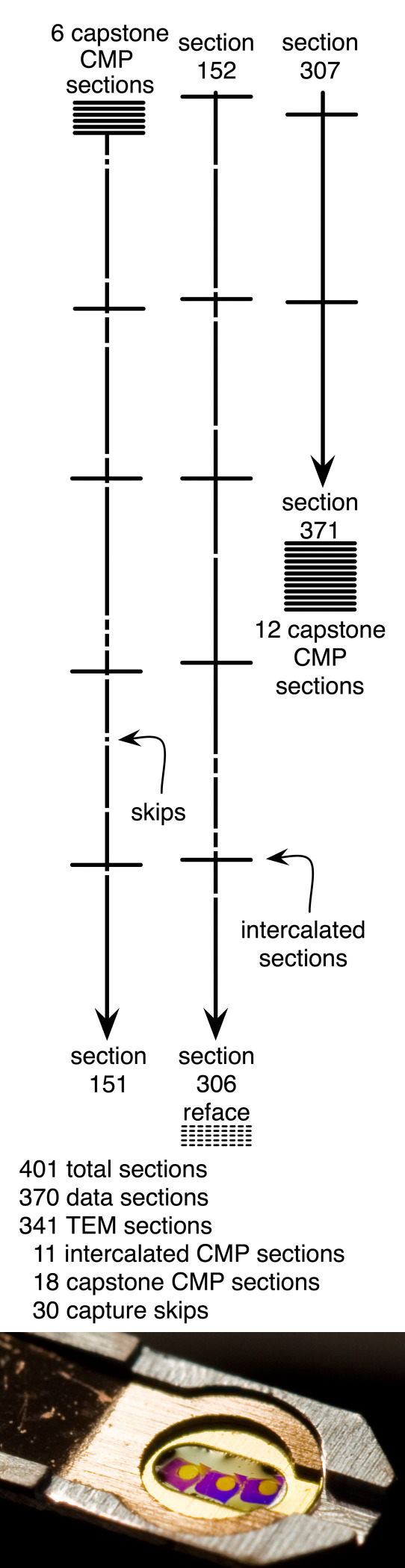
The vertical bars represent section structure of the 0.25 mm diameter column of 341 transmission electron microscope (TEM) data set slices in volume RC1, imaged at 2 nm resolution. The horizontal bars represent capstone and individual intercalated computational molecular phenotyping (CMP) sections for molecular tagging. CMP images were captured at 70 nm/pixel and upsampled to 2.18 nm/pixel in Viking. The CMP skips in the TEM sequence were intentional and created no problems in process tracking. The gaps indicate unplanned capture skips in due to defects that prevented imaging. A block refacing event at section 306 caused a significant (350–400 nm) loss in the ganglion cell layer. At bottom, a single grid carrying three imaged sections is shown. The gold spot on each section indicates the area captured, each spot averaging over 1,000 individual captures at a magnification of 5,000×.

We assembled connectome RC1 for the rabbit retina by combining ATEM imaging [3], computational molecular phenotyping (CMP) [5,8], and excitation mapping using 1-amino-4-guanidobutane (AGB), a channel-permeant organic cation [8-12]. As summarized in Figure 1, a 0.25 mm diameter, 370 serial-section tissue column [3] spanning the inner nuclear, inner plexiform, and ganglion cell layers was imaged by ATEM at a resolution of 2.18 nm/pixel, yielding over 350,000 image tiles in a 16.5 terabyte volume captured over five months at 3,000 images/day. This stage represents the transition from a section (a structure produced by microtomy) to a slice: an image plane computed from a selection of section image tiles. Tiles were automatically mosaicked into slices and slices automatically aligned into a volume using the NCR Toolset [3,13]. The volume was bounded by CMP data sets probed for glutamate, glutamine, glycine, taurine, 4-aminobutyrate (GABA), and the excitation marker AGB. The image column was also intercalated with molecular markers by using every 30th section in the series for CMP. These channels were aligned with TEM imagery to classify neurons, glia, and microglia.

In this paper we introduce the structure and basic features of RC1, demonstrate our data mining strategy, and summarize our initial findings. The basic finding is that RC1 contains biologic data to be mined at several levels, including the three-dimensional (3D) forms of cells, their identities and partners, their molecular phenotypes, their activities in response to stimulation, and their subcellular histoarchitectures. The connectivity of RC1 is being explored with the Viking viewer [3,4], which allows users to view, query, and annotate using conventional network connections. Though it will take many years and >10^6^ annotations to adequately mine RC1, many novel findings have already emerged in our initial 300,000 annotations. This includes the findings that AII amacrine cells receive direct ribbon input from wide-field ON cone bipolar cells; that cone bipolar cell axons are sites of signal integration; and that complex chains of amacrine cells are common network elements.

## Methods

### Excitation mapping, tissue harvesting, and processing

The retinal sample for ATEM image volume RC1 was taken from a light-adapted female Dutch Belted rabbit (Western Oregon Rabbits, Philmath, OR) after in vivo excitation mapping [9,11,12]. All protocols were in accord with the Institutional Animal Care and Use protocols of the University of Utah, the Association for Research in Vision and Ophthalmology (ARVO) Statement for the Use of Animals in Ophthalmic and Visual Research, and the Policies on the Use of Animals and Humans in Neuroscience Research of the Society for Neuroscience. Unless noted otherwise, all chemical reagents were purchased from Sigma-Aldrich Corp. (St. Louis, MO). The animal was tranquilized with intramuscular ketamine/xylazine and deeply anesthetized intraperitoneally with 25% aqueous urethane. The eye was topically anesthetized with 1% lidocaine in 0.1% NaCl 10 min before intravitreal injection with 0.1 ml of 130 mM AGB sulfate with a 23 gauge pressure relief needle at the limbus. The rabbit was positioned between two LCD computer monitors and exposed to 90 min of flickering 3 Hz square wave stimulation of 50% duty cycle in a pattern of one blue and three yellow pulses. There was a corneal flux density of 9.1×10^3^ quanta/sec/cm^2^ at 440 nm for the blue stimulus, dual peaks of 12.5×10^3^ quanta/sec/cm^2^ at 540 nm and 11.6×10^3^ quanta/sec/cm^2^ at 620 nm for the yellow stimulus. The rabbit received a final urethane overdose and was euthanized by thoracotomy in accord with University of Utah Institutional Animal Care and Use Committee guidelines. The eyes were then immediately injected with 0.1 ml fixative with an 18 gauge needle pressure relief, enucleated, hemisected, and fixed for 24 h in 1% formaldehyde, 2.5% glutaraldehyde, 3% sucrose, and 1 mM MgSO_4_ in 0.1 M cacodylate buffer, pH 7.4. The eyes were dissected and isolated retinal pieces were osmicated for 60 min in 0.5% OsO_4_ in 0.1 M cacodylate buffer, processed in maleate buffer for en bloc staining with uranyl acetate, and processed for resin embedding as previously described [14]. The popular osmium-ferrocyanide method for enhancing TEM image contrast was not used [15], as it quenches the immunoreactivity necessary for small molecule mapping. Retinal pieces were remounted in resin for serial sectioning in the horizontal plane through the inner nuclear layer (INL), IPL, and ganglion cell layer [16,17]. For practical reasons (data set size, capture time, and storage costs), we initiated the assembly of RC1 in the mid-INL. Future data sets will span the entire outer plexiform layer–ganglion cell layer volume. Serial sections were cut at 70–90 nm on a Leica UC6 ultramicrotome onto carbon-coated Formvar® films supported by gold slot grids. Two or three sections were placed on every grid. For volume RC1, optical thin sections were captured on multi-spot Teflon®-coated slides (Cel-Line; Erie Scientific Inc.), and processed for CMP [3] as previously detailed and summarized briefly here. After sodium ethoxide etching, the sections were probed with antihapten IgGs targeting AGB, GABA, glycine, glutamate, glutamine, or taurine (Signature Immunologics Inc., Salt Lake City, UT) and visualized with silver-intensified 1.4 nm gold granules conjugated to goat antirabbit IgGs (Nanoprobes, Yaphank, NY). Optical (8-bit 1388 pixel x 1036 line frames) images were captured, mosaicked, aligned, and processed for classification using isodata clustering and principal components analysis [3]. The RC1 volume was initiated and terminated with the 10-section optical capstone CMP series and intercalated every 30 sections with one CMP section (Figure 1).

### Volume assembly

RC1 was created as described in Anderson et al. (2009) [3] and summarized here. The center of a canonical field 250 μm in diameter was identified in each grid using SerialEM [18] and captured as an array of image tiles at roughly 950–1100 tiles/slice with 15% overlap. The capture took five months at 3,000 images daily, yielding over 350,000 individual captures and over 16.5 terabytes of active storage. We have since improved capture rates to achieve 5,000 images daily. The NCR Toolset was used to generate mosaics and volumes [3,13]. SerialEM metadata position information used by the NCR Toolset application ir-translate produces precise initial image mosaics which are refined by ir-refine-grid to correct for image aberrations. Slice-to-slice TEM image registration is automated by ir-stos-brute and ir-stos-grid. CMP-to-TEM registrations are operator-guided with ir-tweak.

### Volume integrity

RC1 was manually sectioned and stained and contains common defects such as skipped imaging sections, folds, cracks, and stain artifacts. A complete summary of the library is publicly available at Science-Connectome and is diagrammed in Figure 1. The data set is composed of 370 sections: 341 TEM sections, 18 capping CMP sections, and 11 intercalated sections for CMP spaced 30 sections apart. In retrospect, we would have placed them more frequently, as skipped sections were mostly inconsequential and the embedded molecular data were very useful. There were 30 captures skipped either due to loss of a Formvar® film or section distortion preventing imaging. Importantly, tracing is effective even with skips and defects, as most cells, processes, synaptic terminals, and even synapses and gap junctions extend well over 100 nm and an occasional skip or occlusion usually poses little difficulty. Section defects (folds, dirt) are rarely aligned from section to section. In some instances when processes are smaller than 100 nm in diameter (e.g., intervaricosity neurites in amacrine cells), defects can lead to the loss of tracking. In many cases, these lost elements can be recovered as the other processes in a region are tracked and assigned, since every process in transit must have a source and target. One serious loss of five sections (350–400 nm loss) happened near the end of the series near the bottom of the IPL, where the bloc had to be refaced to maintain section stability. Even so, most processes from most cells, including ganglion cells, were traceable. On balance, manual sectioning is effective in forming connectome data sets. Improvements may be achieved with automated sectioning, but the absence of automated section tools should be no barrier to connectomics research.

### Image viewing and annotation

RC1 was viewed and annotated with Viking, originally called NGVV in Anderson et al. [3,4]. In developing Viking, we realized that we did not need full volume 3D viewing, but rather the ability to display and annotate one slice at a time with guidepost markers from annotations above and below, paging through the data like a book. The Viking viewer is based on dynamically applying the computed slice-to-slice transforms to the image region desired by the user. The advantages and details of this implementation are available in Anderson et al. [4]. On startup, Viking points to a website containing the desired volume data and generates slice-to-volume transforms for each slice. Multiple users concurrently annotate RC1 and the annotation database is stored on a Microsoft SQLExpress server and exposed via HTTP. Users navigate and annotate with keystroke, mouse, and menu options. While very rich neural ontologies have already been developed [19,20], initial annotation is best done with a small set of markup tags, as these can later be translated to richer schemata. Our annotation method was designed to be flexible and not restricted to circuitry, allowing users to define their own ontologies. Various visualizations are achieved via applications that abstract the required information for a set of cells in the database. For example, 3D renderings are managed by VikingPlot, a compiled Matlab application that queries structure information from the annotation database and renders surfaces for display. A more practical tool is graph visualization of network topologies for analysis, novel pathway discovery, and error detection and correction; these are all achieved via a web-services strategy. Again, this approach is detailed in Anderson et al. [4]. Viking also integrates CMP and TEM data, allowing the correlation of cells and processes with specific molecular signals. In particular, we tag individual amacrine cells as γ+ (GABA+) or G+ (glycine+), certain bipolar cells as G+ BCs (these all turn out to be ON cone bipolar cells), and certain ganglion cells as γ+ GCs, likely reflecting heterocellular coupling with amacrine cells.

### Reimaging

Reimaging is done for three reasons: improved resolution, proper tilt, and expanding connectome volumes to track off-edge cells. Some structures such as gap junctions required imaging at a much higher resolution than 2 nm to validate contacts. Similarly, some (most) synapses were not normal to the plane of slice and, while both presynaptic vesicle clusters and postsynaptic densities remain obvious and distinctive, the synaptic cleft is often indistinct. Goniometric tilt can retrieve those images. Finally, some processes run off the edges of the captured primary volume and small secondary wings could be captured. Regions targeted for reimaging were identified by their data set coordinates and low to high nested magnification images captured to guide the TEM analyst. High resolution (20,000–60,000×) and goniometric tilt series were taken both digitally and on film for optimal resolution and bit-depth, and scanned at 16 bits at 2,540 dpi on a Kodak (Creoscitex) iQSmart2 prepress flatbed scanner. Extensions of capture volumes overlapped the existing volume and were captured as described previously.

### Data sharing

The entire RC1 data set is available at Connectome, as are the associated analytical tools. Thus, our data summaries or interpretations can be explored by anyone. The software resources for this project are available as free (SerialEM) or open-source (NCR Toolset) applications or via a free license (Viking and web-services tools) for educational use through the University of Utah. The RC1 data set is also available through a free educational license on user-provided storage media.

### Image preparation

The procedures for preparing publication figures from raw image data followed those detailed in Anderson et al. [3]. All of the raw optical image data are available upon request and RC1 is public access. Multimodal registered optical images were max-min contrast stretched and sharpened using unsharp masking at a kernel extent of roughly 540 nm. CMP data sets were displayed as mixtures of classical 8-bit red-green-blue (RGB) and cyan-magenta-yellow channels depending on complexity. For example, pure molecular triplets were mapped as RGB sets, doublets usually as green-magenta (where magenta M=R+B), and single channels in various overlay colors. TEM images from the NCR Toolset process have high contrast and were softened by adjusting the gamma to 1.2–1.3. Contrasts were adjusted to match brightness histograms across slices and images were sharpened using unsharp masking with 1–3 pixel kernels (2–6 nm). Overlay methods for combining optical and TEM images computed new hue, saturation, and brightness triplets for a new image using the TEM grayscale brightness and hue and saturation from the RGB optical image or overlay color. Occasionally, fourth or fifth channels were added using standard alpha blending [21]. Renderings of structures in Viking were created in Matlab (Mathworks Inc., Natick, MA) 2009a. The annotation system stores a point and a radius to describe the largest circle that can fit inside a cell on each slice. Annotations are linked to their neighbors on adjacent slices with a graph structure. To render cells, we drew a cylinder between each pair of linked annotation circles using the Matlab patch function and Phong lighting. Annotation circles with only two links were tilted from the XY plane at an angle one half of the total angle between the linked locations on the axis normal to the plane described by all three annotations. Circles were chosen because of the speed of the annotation user interface. As a result, the renderings are an approximation of the cell, but correctly capture the dendritic morphology available from the EM volume.

## Results

We here describe the organization of the RC1 volume and its integrated molecular markers, as well as aspects of our explorations of RC1 that expand our views of retinal networks. Those expansions include data mining of mammalian AII amacrine cells, recapitulating their well known features, exposing novel synaptic associations, and identifying more synaptic partners. We also provide a quantitative analysis of amacrine cell synapses onto and noncanonical output ribbon synapses from the descending axons of bipolar cells. Amacrine cells pose one of the most difficult challenges in connectomics because of their large arbor sizes and diverse forms. We provide an example of one way to begin mining amacrine cell populations by discovering complex synaptic chains and working backwards to their sources. All of these analyses include intrinsic molecular markers as part of the cell identification process. We also show preliminary evidence that extrinsic activity reporters can be correlated with specific cell classes at the TEM level.

### The organization of RC1

**Figure 2:**
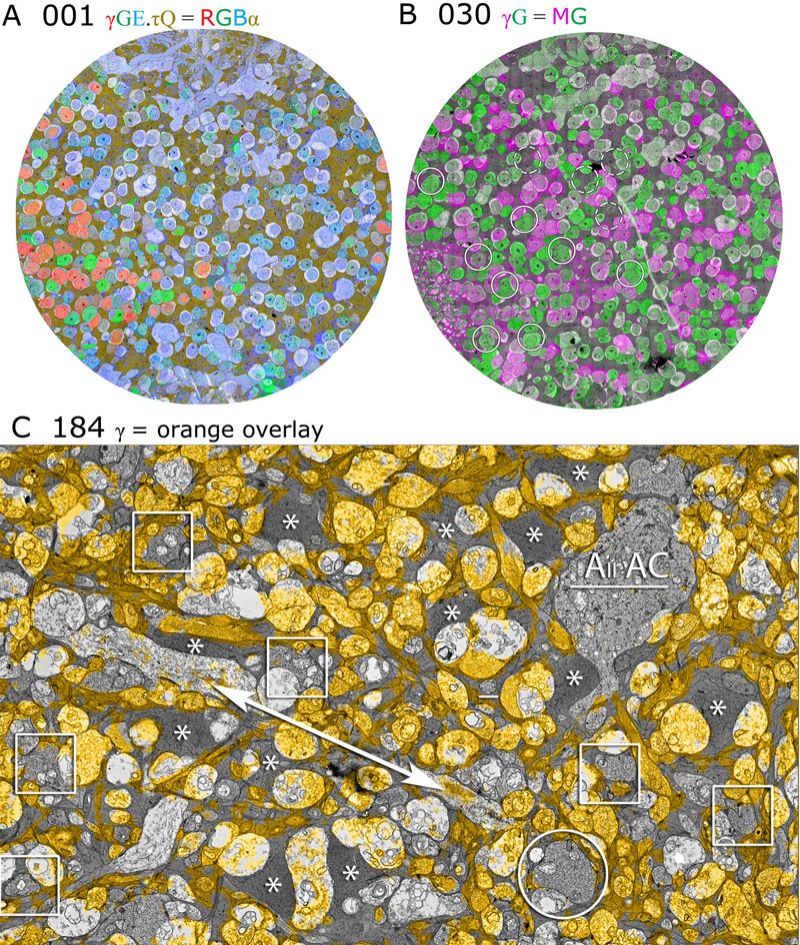
Connectome RC1 data sets were visualized by fusing transmission electron microscope (TEM) images and computational molecular phenotyping (CMP) signals. **A**: TEM section 001 is a near-horizontal plane section through the inner nuclear layer (INL) of the retina, visualized with 4-aminobutyrate(GABA).glycine.glutamate (γGE) → red.green.blue (RGB) transparency mapping, displaying retinal neurons, and a dark gold alpha (α) channel derived from taurine and glutamine (τQ) channels marking retinal glia (γGE.τQ) → RGB.α (see Methods). GABA+ (red) neurons are amacrine cells, while glycine+ (green) neurons are either amacrine or an ON cone bipolar cell subset. Glutamate+ (blue) neurons are largely bipolar cells. The image width is 243 μm. **B**: TEM section 030 is a connectome slice roughly 2.5 μm deeper in the INL, visualized with a GABA.glycine → magenta.green transparency (γG=MG; see Methods). The circled cells represent 12 validated AII amacrine cells, 8 visible in section 030 (solid circles) and 4 originating in a plane beneath section 030 (dashed circles). The image width is 243 μm. **C**: TEM section 184 with orange GABA (γ) overlay (see Methods) shows that all bipolar cell terminals are GABA- (boxes), as are lobular appendages of AII amacrine cells (circle), a descending portion of AII amacrine cell C4835, and the radial fibers of Müller cells (asterisks). Numerous GABA+ processes and a weakly labeled ganglion cell dendrite (arrow) are present. The image scale is 5 μm.

**Figure 3:**
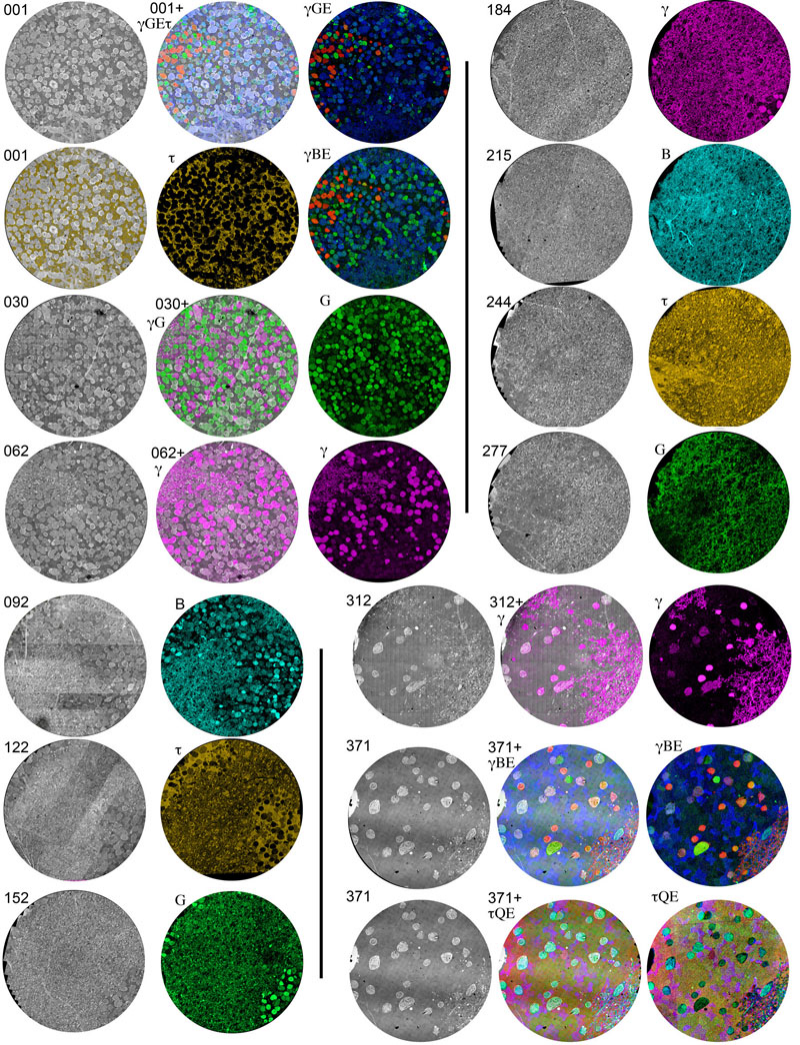
The computational molecular phenotyping (CMP) matrix for volume RC1 is bounded by CMP data sets and intercalated every thirty sections with ultrathin CMP sections that map molecular tags onto transmission electron microscope (TEM) data. Each row of 2 or 3 fields contains a TEM slice with its associated index number, one or more optical CMP channels composed of one to four molecular tags, and for fields in the inner nuclear and ganglion cell layer, overlay images of the CMP data registered onto the TEM channel. Each disc is 243 μm in diameter. The matrix was assembled from 32x down-scaled TEM data sets (70 nm/pixel). This represents a threefold oversampling of the optical data. The abbreviations and color key for the figure are: B, 1-amino-4-guanidobutane, color=cyan; E, glutamate, color=blue; G glycine, color=green; γ, GABA, color=red (slices 001, 371) or magenta (slices 62, 184, 312); τ taurine, color=gold (slice 001), red (slice 371), or orange (slices 122, 244); γG → γ magenta: G green; γGE → γ red, G green, E blue; γGEτ → γ red, G green, E blue + gold τ alpha channel overlay mask; γBE → γ red, B green, E blue; τQE → τ red, Q green, E blue.

Data set RC1 contains 284 bipolar cells, 167 GABA-positive (γ+), and 118 glycine-positive (G+) amacrine cells, over 350 Müller glia, 18 validated ganglion cell somas (with many more ganglion cell dendrites crossing the volume), 19 horizontal cells, and at least 76 microglia. CMP mapping allows us to visualize each cell’s molecular classification independent of tracing, annotation, and reconstruction. Figure 2 demonstrates the fusion of CMP data with TEM imagery of RC1 for slices 001, 030, and 184 of the data set. TEM slice 001 (Figure 2A) was preceded by a capstone of CMP data and is partitioned by AGB (activity), GABA, glycine, glutamate, glutamine, and taurine signals into distinct cell populations. Further segmentation of RC1 by Principal Components Analysis (PCA) and K-means clustering [3,8] yields 35 molecular classes or superclasses of cells: 2 horizontal cell classes, 11 bipolar cell classes, 10 γ+ amacrine cell classes/superclasses, 5 G+ amacrine cell classes/superclasses, at least 7 ganglion cell superclasses, 1 Müller cell class, and 1 microglial cell class. These will be described in future work. As the volume was assembled, intercalated CMP channels were mapped onto adjacent TEM sections, each allowing direct segmentation of the TEM data. Slice 001 is centered on the bipolar cell layer, dominated by glutamate-rich (blue) signals, with a portion of the amacrine cell layer exposed at the lower left edge. Slice 030 is some 2.5 μm deeper in the volume and is dominated by γ+ (magenta) and G+ (green) amacrine cells (Figure 2B). A portion of slice 184 (Figure 2C), ≈15 μm deeper, is shown at a higher resolution, demonstrating that mapping of GABA signals onto TEM imagery of single processes permits the discrimination of strongly γ+ amacrine cells, weakly γ+ coupled ganglion cell processes, and GABA negative elements such as glia, AII amacrine cells, and bipolar cells. Thus, the molecular signatures of network elements can be identified at the synaptic level. Figure 3 summarizes the full CMP library throughout RC1 and is used to identify cells and processes in the volume.

### Building extended AII amacrine cell networks

**Figure 4:**
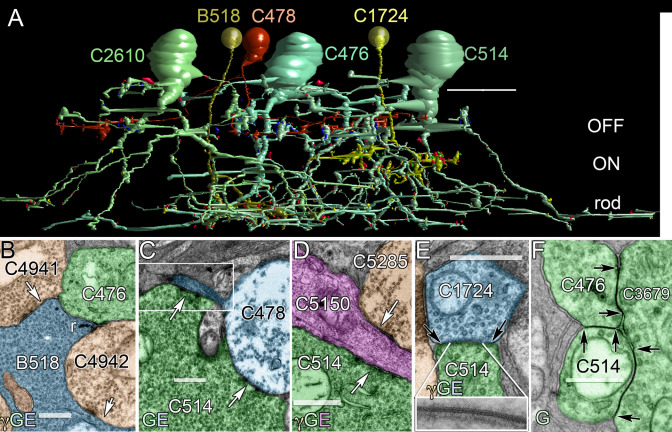
A fragment of the mammalian AII amacrine cell network is visualized by rendering and transmission electron microscope (TEM). **A**: Viking-rendered AII amacrine cells (C476, C514, C2610) and rod (B518), OFF (C478) and ON (C1724) bipolar cells form a local network. Each bipolar cell was chosen to mark the center of the cone OFF, cone ON and rod driven zones of the inner plexiform layer. The small red, blue and yellow details represent postsynaptic, presynaptic and gap junction contact sites. They are scaled to true size, so most of them are below the figure’s resolution. Only the largest are visible (scale, 20 μm). **B**: Rod BC B518 (blue) presynaptic (r) to AII amacrine cell C476 (green) and γ+ AC C4942 (orange); γ+ ACs C4941 and C4942 are presynaptic (arrows) to B518. **C**: AII amacrine cell C514 (green) to OFF cone BC C478 (blue) synapses (arrows). C514 makes conventional synapses onto C478 at its terminal swelling and fine inter-varicosity processes (box, 6 sections away). **D**: AII amacrine cell C514 (green) and γ+ AC C5285 (orange) are both presynaptic (arrows) to OFF GC C5150 (magenta). **E**: Heterocellular coupling (between black arrows) between ON cone BC C1724 (blue) and AII amacrine cell C514 (green). The inset (width 169 nm) is a high resolution tilt TEM image of the gap junction. **F**: Homocellular coupling (arrows) occurs among AII amacrine cells C514, C476, and C3679. The scales for panels **B**-**F** are 500 nm.

**Figure 5:**
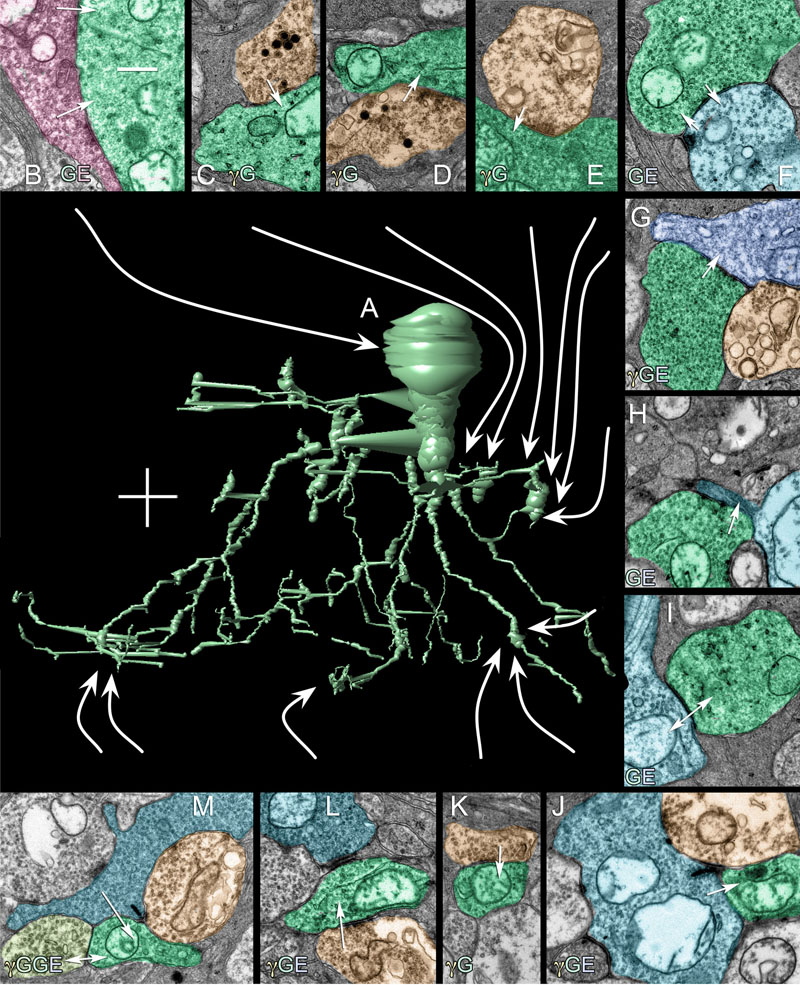
Numerous synaptic connections converge on AII amacrine cell C514. **A**: The central image is a 3D VikingPlot rendering of C514 (scale, 10 μm). Surrounding the cell are instances of different synaptic connections made by C514. In each panel, green profiles are C514, orange profiles are γ+ ACs, azure profiles are BCs, blue profiles are GCs, red profiles are γ-, G- and glutamate+. Arrows indicate direction of synaptic signaling and double arrows indicate gap junctions. **B**: C514 is postsynaptic to large γ-/G- axosomatic synapses likely deriving from TH1 (tyrosine hydroxylase immunopositive type 1) cells. However, the architecture of the synapse is of a fast conventional transmitter, likely glutamate (see Figure 7 and Figure 8). **C**: C514 is postsynaptic to γ+ / peptidergic processes in the OFF sublayer at points where dense-core, peptide vesicles form fusion complexes. **D**: C514 is postsynaptic to γ+ / peptide processes in the OFF sublayer at a conventional inhibitory synapse. **E**: C514 is postsynaptic to conventional, non-peptide γ+ processes in the OFF sublayer. **F**: C514 is both presynaptic and postsynaptic to an OFF cone bipolar cell. **G**: C514 is presynaptic to an OFF ganglion cell. **H**: C514 is presynaptic to an OFF bipolar cell. **I**: C514 is coupled to an ON cone bipolar cell. **J**: C514 is postsynaptic to an ON cone bipolar cell. **K**: C514 is postsynaptic to a γ+ amacrine cell. **L**: C514 is postsynaptic to a γ+ type AI amacrine cell. **M**: C514 is postsynaptic to a rod bipolar cell and coupled to another AII amacrine cell. The scales for panels **B**-**M** are 500 nm.

Our first goal was to validate our technique against the gold standard for neural reconstruction: the AII amacrine cell [15,22]. If our analyses could not rapidly recapitulate basic prior findings, they would not be useful tools to discover new networks or extend models for physiology. Figure 2B displays RC1 slice 030 and the locations of 12 G+ AII amacrine cells validated by connectivity and molecular signatures. Our complete analysis of all these cells is beyond the scope of this paper, but here we summarize both previously known and new features of the AII amacrine cell system. We reconstructed 4 AII amacrine cells to near completion and 11 other cells are partially complete. For these four cells, additional small processes are sometimes found by remapping completed regions, but the incremental addition is slow, suggestion substantial completion. The four cells are about 1 mm ventral to the visual streak and have maximal arboreal dendritic diameters of 67±6 μm (mean±1 standard deviation [SD]). They showed 275±56 postsynaptic sites, 70±12 presynaptic sites, and 76±17 gap junction sites. More importantly, the postsynaptic/presynaptic ratios (4±0.4) and postsynaptic/gap junction ratios (3.6±0.3) for the same cells have a twofold smaller coefficient of variation than the raw counts, suggesting that we have sampled the contact types proportionally in each cell. As summarized in Figure 4 and Figure 5, the dendrites of AII amacrine cells span most of the IPL and contact members of each major superclass of bipolar cells in unique patterns. At present, we have identified over 15 types of bipolar cells, largely but not completely corresponding to the schema of MacNeil et al. (2004) [23]. That data set will be the subject of future papers. AII amacrine cells are purely postsynaptic to rod bipolar cells; coupled to cone ON bipolar cells via relatively large gap junctions; and are both presynaptic and postsynaptic to cone OFF bipolar cells, as previously described [15]. Our new data support and extend those findings. Figure 4A shows automated Viking renderings of four identified classes of bipolar cells (rod, ON cone, wide-field ON cone, and OFF cone) connected to three neighboring G+ AII amacrine cells. Functionally, the AII pathway aggregates rod bipolar cell signals (Figure 4B) and distributes them into OFF cone pathways by chemical synapses (Figure 4C,D) and ON cone pathways by gap junctions (Figure 4E). The coupling of ON cone bipolar cells to AII amacrine cells also generates a distinctive ON cone bipolar cell signature via glycine leakage [24-27] visible in our CMP data. Of the bipolar cells that terminate in the nominal ON sublayer, those with gap junctions onto AII amacrine cells are all G+. This association is quantified below.

**Figure 6:**
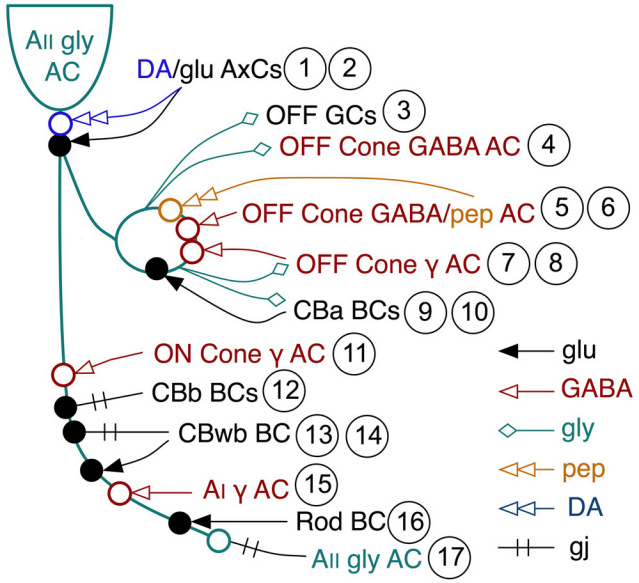
Twelve different neuronal classes generate at least seventeen distinct input-output motifs involving AII amacrine cells: 1, 2 represent the dopamine and glutamate inputs from TH1 axonal cells (AxCs); 3 and 4 are outputs to OFF ganglion cells (GCs) and OFF γ+ cone amacrine cells (AC); 5,6 are inputs from dual GABA/peptide amacrine cells; 7,8 is output to another class of γ+ amacrine cells; 9,10 are outputs to and inputs from OFF cone CBa bipolar cells (BCs); 11 is input from ON cone γ+ amacrine cells; 12 is coupling to several classes of ON cone CBb bipolar cells; 13 and 14 are coupling and ribbon inputs from CBwb BCs; 15 is input from AI amacrine cells; 16 is input from rod bipolar cells; 17 is coupling with other AII amacrine cells.

**Figure 7:**
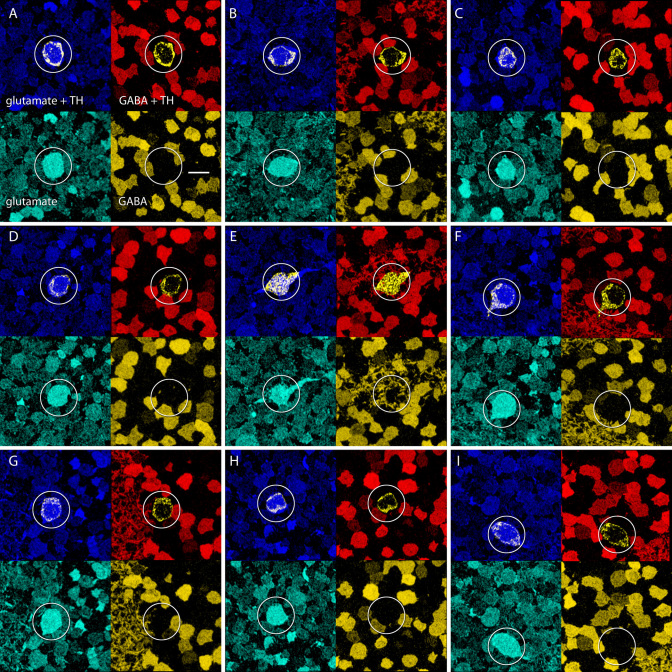
TH^+^ (tyrosine hydroxylase immunopositive) cells have glutamatergic, not GABAergic signatures. The nine panels show nine TH^+^ cells from a single rabbit retina (**A**-**I**), probed for TH, glutamate and GABA in serial 200 nm sections. Each panel shows four mappings: upper left TH (yellow) + glutamate (blue), upper right TH (yellow) + GABA (red), lower left glutamate alone (cyan), lower right GABA alone (yellow). The location of each TH^+^ cell is circled. Each TH^+^ cell has a glutamate signal higher than the surrounding amacrine cell somas and equivalent to that of a ganglion cell. TH^+^ cells have no measurable GABA signal. Scale, 10 μm.

**Figure 8:**
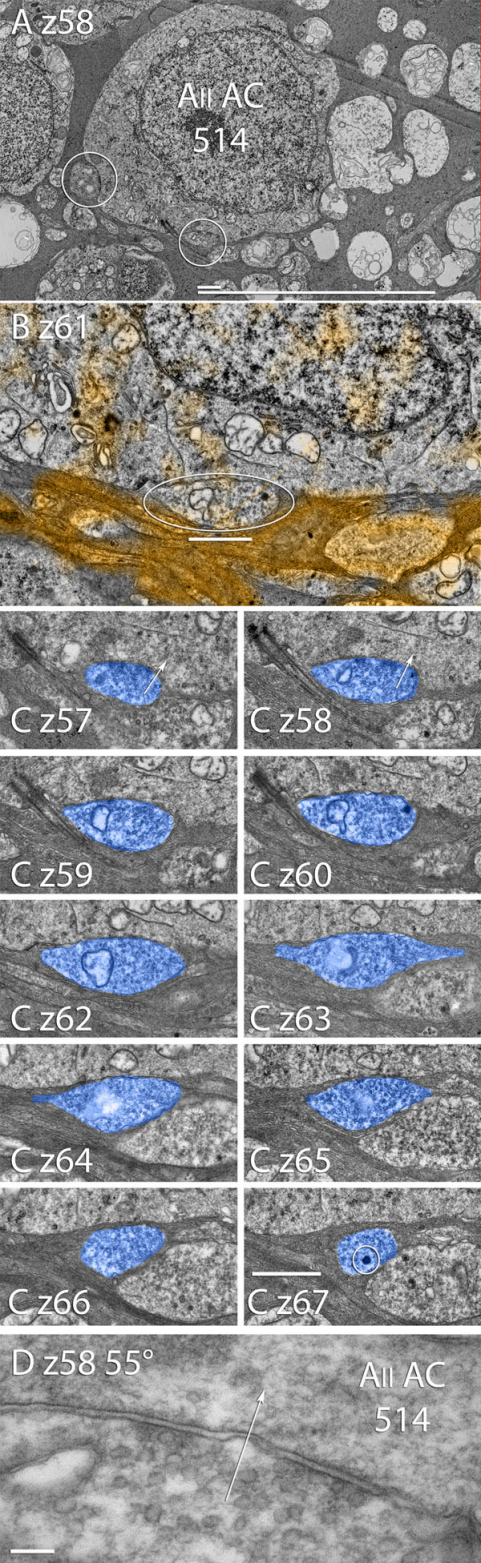
AII amacrine cell 514 in RC1 displays axosomatic synaptic input. **A**: Section 58 (z 58) shows two axosomatic synapses (circled, Scales, 10 and 1 μm). **B**: Section 61 (z 61), with GABA overlay in orange, shows that the axosomatic synaptic terminal has the same GABA negative signal as the AII amacrine cell but is flanked by orange GABA+ processes (Scale, 1 μm). **C**: A serial section series from section 57 (z 57) through 67 (z 67), omitting section 61 shown in panel **B**, shows that axosomatic synapses are formed at z 57 & z 58. Several sections show large dense-core vesicles (circle in z 67) characteristic of TH cells (Scale, 1 μm). **D**: Goniometric tilt re-imaging of the oblique synaptic contact in panel C z 58. A 55° tilt aligns the axosomatic contact membrane, and clearly shows a characteristic 10 nm synaptic gap and polarity (arrow) targeting AII amacrine cell 514.

The range of connections made by AII amacrine cells is summarized in Figure 5 and Figure 6 for AII amacrine cell 514. Each AII amacrine cell engages in no fewer than 17 kinds of interactions. This includes somatic synapses onto AII amacrine cells, which suggests both indirect dopaminergic [28] and direct glutamatergic inputs [29] from TH1 axonal cells (Figure 7, Figure 8); extensive gap junctions between pairs of AII amacrine cells and between AII amacrine cells and G+ ON cone bipolar cells; inputs from at least five different classes of γ+ amacrine cells at every level of the IPL and a possible peptidergic input; well known ribbon synapses from OFF cone bipolar cells and rod bipolar cells; and extensive synaptic output from AII amacrine cells on the OFF layer, including OFF cone bipolar cells, γ+ amacrine cells, and validated ganglion cell targets. AII cells may target more than one class of OFF ganglion cells, but it is also clear that certain ganglion cells that branch in the OFF layer receive OFF bipolar cell inputs but do not receive any AII cell inputs. Finally, a new dimension of AII networks emerged. AII amacrine cells receive direct synaptic ribbon inputs from G+ wide-field ON cone bipolar cells (Figure 5J). They do not receive ribbon inputs from any other class of ON cone BCs.

**Figure 9:**
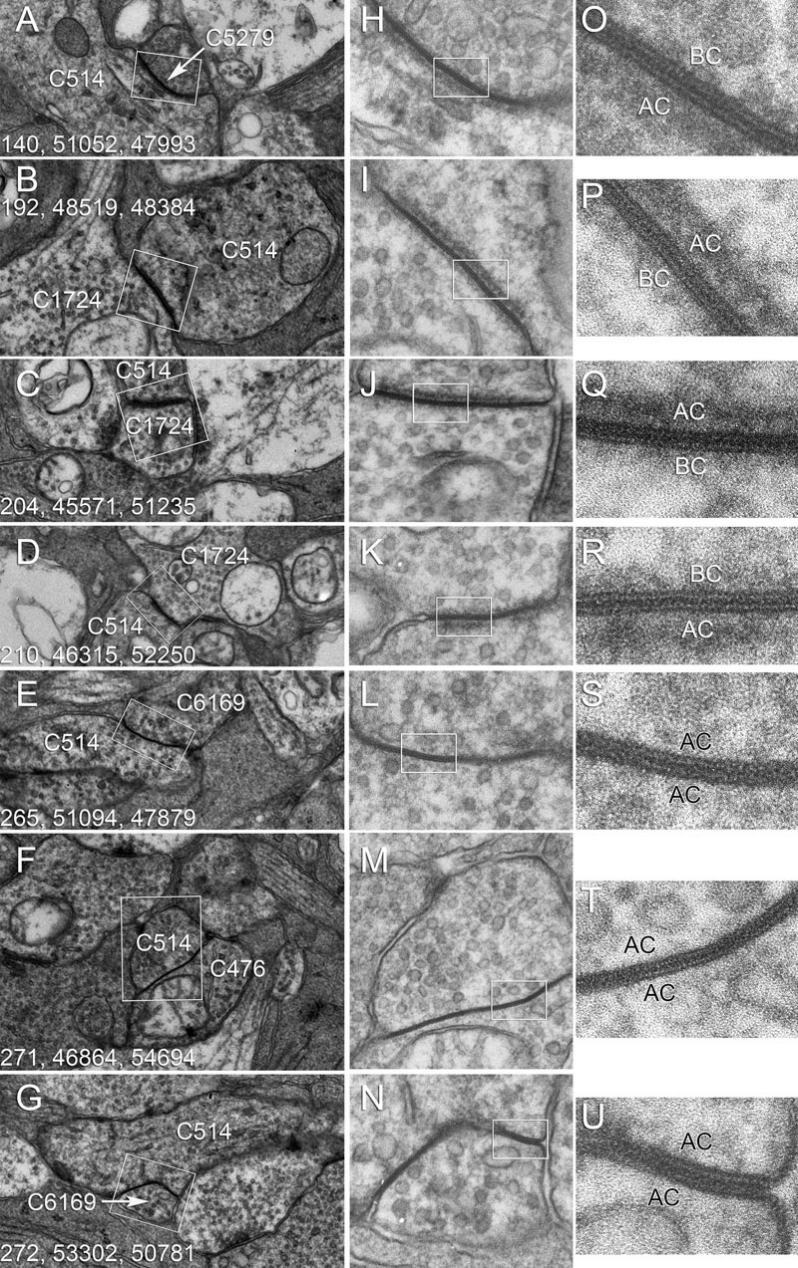
Gap junctions between identified cells are visualized by transmission electron microscope (TEM) in the RC1 data set and after re-imaging at 0.3 nm resolution. Panels **A**-**G** are native Viking images showing putative gap junctions (box areas). Each panel is 3570 nm wide. The numbers denote the location of the image in the RC1 image volume (section number, x location, y location). Panels **H**-**N** are the same locations re-imaged at 40,000× on Kodak 4498 Electron Microscopy Film and digitized at 2540 dpi and 16-bits. Each panel is 602 nm wide. Panels **O**-**U** are validated gap junctions scaled from the boxed regions in **H**-**N**. Each panel is 150 nm wide.

**Figure 10:**
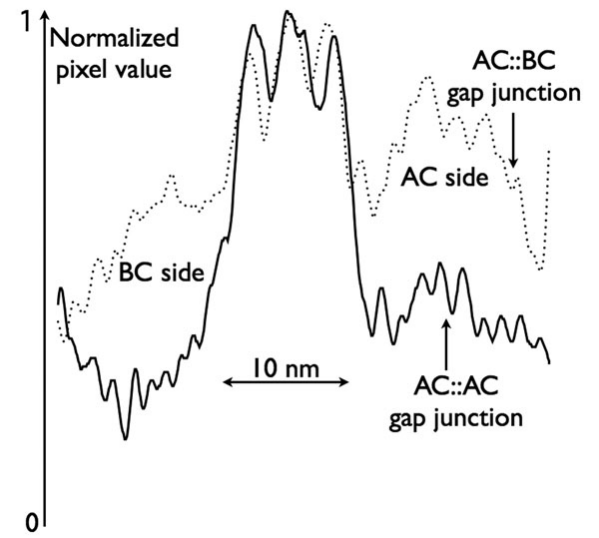
Gap junctions can be displayed as transmission electron microscope (TEM) image density profiles for homocellular (black lines) and heterocellular (dotted lines) pairings. A central 12–13 nm wide pentalaminar zone with three sharp dense bands representing membrane protein density is common to all retinal gap junctions. But the flanking cytoplasmic compartments differ markedly. Homocellular gap junctions between pairs of AII amacrine cells show little protein density near the membrane. Conversely, heterocellular gap junctions between AII amacrine cells and cone bipolar cells show thick bands of protein density extending about 10 nm and over 15 nm from the bipolar cell and amacrine cell faces, respectively.

AII amacrine cells form extensive homocellular and heterocellular coupling networks. While we cannot visualize membrane bilayers with our 2 nm resolution, gap junctions are nevertheless distinctive in RC1 as uniquely fused dense membranes (e.g., Figure 4F, Figure 5I) and we can re-image any region of the sample at higher resolution as needed. In a very short time, analysts can readily recognize even very oblique gap junctions, as synapses and nonspecialized membranes never present a homogeneous band of high electron density between cells (e.g., Figure 4E), even at very high tilts. Figure 9 summarizes a collection of heterocellular gap junctions between cone bipolar and AII amacrine cells, and homocellular gap junctions between AII amacrine cell pairs, first identified at 2 nm resolution (Figure 9A-G) and then re-imaged at 0.3 nm film resolution with goniometric tilt to optimize gap junction alignment (Figure 9H-N). Every putative gap junction identified at 2 nm has proven to be a valid gap junction when re-imaged. High resolution imaging reveals distinct membrane-associated densities (Figure 9O-U) present only in heterocellular gap junctions. The densities were strongly asymmetric and always thicker on the amacrine cell side of heterocellular junctions (Figure 10).

### Bipolar cell axonal synapses

**Figure 11:**
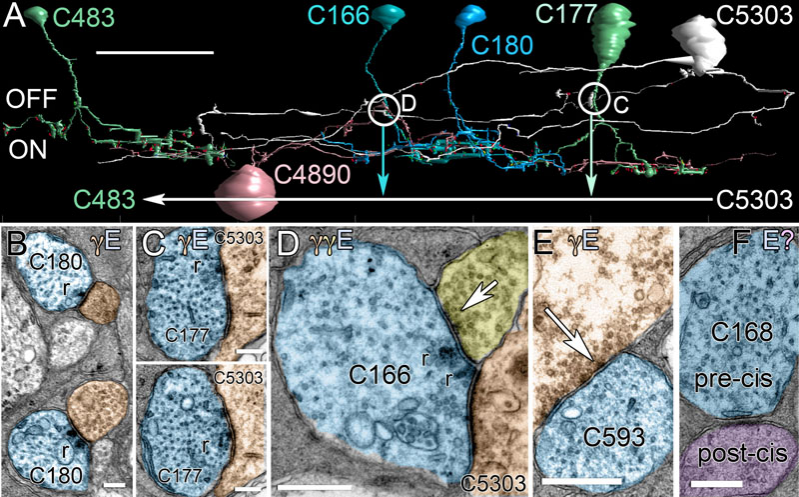
RC1 contains novel retinal networks. **A**: A Viking rendering of γ+ amacrine cell C5303 shows that it is postsynaptic to ON cone bipolar cells at axonal ribbon sites (circles C and D), presynaptic to ON cone bipolar cell C483, and co-stratifies with ON starburst amacrine cell C4890 (scale 20 μm). The circles indicate corresponding transmission electron microscope (TEM) images. **B**: Axonal ribbons (r) from ON cone BC C180 target AC neurites as the axon bifurcates in mid-inner plexiform layer. **C**: ON cone BC C177 makes axonal nanoribbon contacts onto γ+ amacrine cell C5303. **D**: Axonal ribbon synapses from C166 target cell C5303. C166 receives an axonal veto synapse from a yet unidentified amacrine cell (arrow). **E**: A large γ+ amacrine cell makes an axonal veto synapse onto an OFF cone bipolar cell axon. **F**: An axonal cistern contact is formed between ON cone bipolar cell C168 onto an amacrine cell process. E denotes glutamate; γ denotes GABA; and question mark denotes unknown. The letter colors match the profiles in the image. The scales for images **B**-**F** are 500 nm.

**Figure 12:**
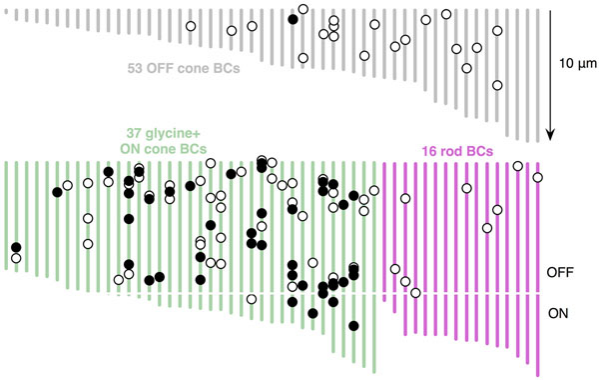
Axonal ribbon synapses (black circles) and veto synapses (white circles) are distributed along the axons of 105 reconstructed bipolar cells. Grey lines are OFF cone bipolar cells terminating high in the inner plexiform layer. Green lines are G+ ON cone bipolar cells. Magenta lines are rod bipolar cells with extensive AI and AII amacrine cell contacts. Each line indicates the length of the axon from its point of entry to its terminal expansion level in the inner plexiform layer (IPL). The physical cells are longer as we show only the axon, not the entire terminal arbor. The break in the lower panel axons represents the approximate position of the lower limit of identified OFF bipolar cell processes. Importantly, some bona fide ON bipolar cells axons are shorter than the longest OFF bipolar cell axons and terminals and they co-mingle.

While building the AII amacrine cell patch, we also began reconstructing ≈200 bipolar cells. While that project is still ongoing, we have reconstructed over 100 bipolar cell primary axons. This revealed unexpected synaptic motifs, suggesting that bipolar cell axons represent an unexpected signal processing compartment. ON cone bipolar cells demonstrate abundant small presynaptic ribbon contacts along their axons (Figure 11A-D). Both ON and OFF cone bipolar cells receive amacrine cell synapses on their axons (Figure 11D,E), and some make novel cistern contacts associated with discrete postcistern densities in target neurons (Figure 11F). Axonal ribbon synapses (Figure 11A-D, Figure 12) are presynaptic specializations composed of predominantly suboptical ribbons (50–200 nm) that aggregate ≈30–50 synaptic vesicles and form synaptic monads or dyads along the axon, including the OFF sublayer of the retina, apparently violating the nominal ON and OFF layering of the IPL. Their dominant targets are the axons of γ+ neurons that course up to 250 μm laterally in the IPL, some synapsing on distant bipolar cell terminal arbors (Figure 11A). Certain ON bipolar cells also target specific non-AII G+ amacrine cells. While the axonal ribbons are usually quite small, the postsynaptic plaques in the target processes are always large, usually at least 500 nm in diameter and often larger, consistent with the ability to detect 2-amino-3-[5-methyl-3-oxo-1,2- oxazol-4-yl]propanoic acid (AMPA) receptor immunoreactivity along bipolar cell axons [30]. Axonal ribbon contacts are made almost exclusively by ON cone bipolar cells (Figure 12). The binomial probability that nanoribbons are randomly distributed across bipolar cell classes is <1.3×10^−6^. However, 3 of 46 ON cone bipolar cell nanoribbons that made conventional bipolar cell contacts in the ON sublayer also contacted bistratified ganglion cells in the OFF sublayer (this will be addressed in future papers).

Most ON and OFF cone bipolar cells and some rod bipolar cells (Figure 11, Figure 12) also receive axonal amacrine cell synapses on their descending axons, many of which we can validate as γ+ (Figure 11D,E). The binomial probability that these synapses are randomly distributed across bipolar cell classes is <5.6×10^−3^. Finally, ON cone bipolar cell axons often form specialized contacts that we term cistern contacts (Figure 11F). Their key features are the withdrawal of Müller cell processes to expose a patch of axon membrane, the placement of a single cistern of membrane resembling smooth endoplasmic reticulum next to the exposed axon region, and a distinctive postcistern density resembling a classic excitatory synapse postsynaptic density in target cells.

### Amacrine cell network complexity

**Figure 13:**
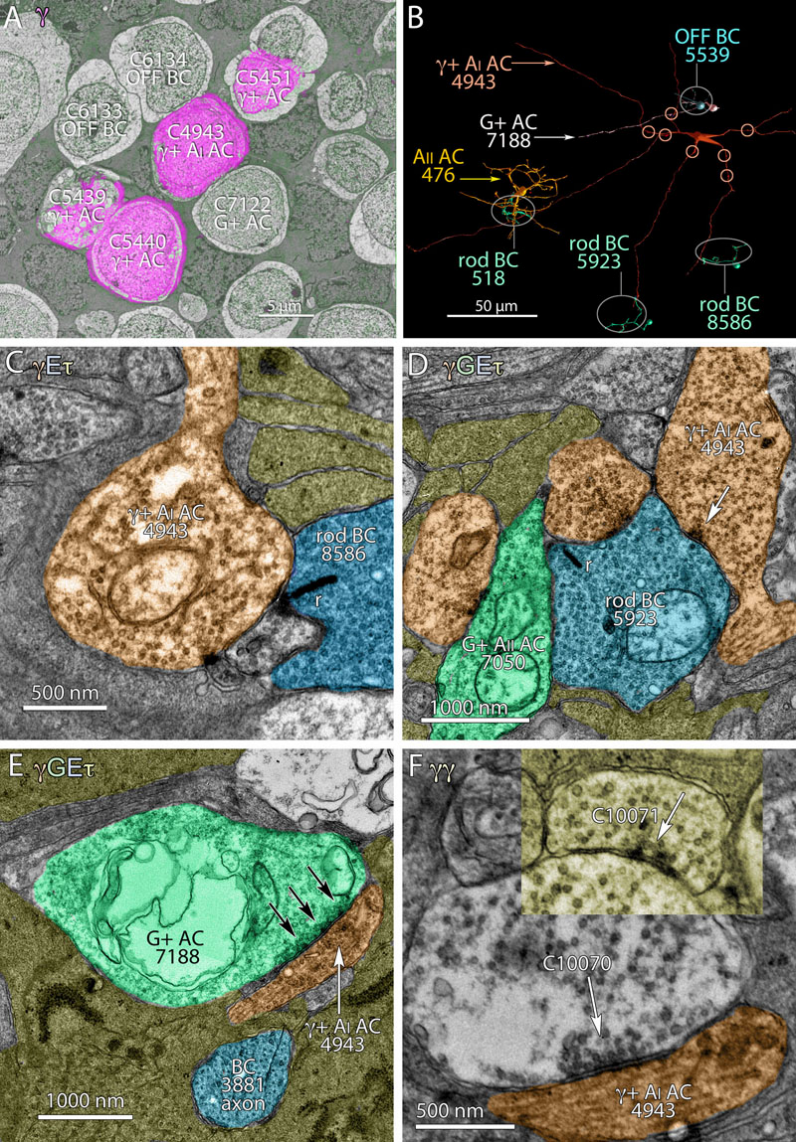
AI and AII amacrine cells display complex networks. **A**: Transmission electron microscope (TEM) section 062 shows γ+ AI AC 4943 and neighboring BCs and ACs (green) with a magenta GABA overlay. **B**: γ+ AI amacrine cell 4943 (red) spans the width of the RC1 volume and some of the cells associated with it are rod bipolar cells 518, 5923, 8586; OFF cone bipolar cell 5539; G+ AII amacrine cell, 476; G+ OFF amacrine cell, 7188. The circles over the proximal dendrites of amacrine cell 4943 denote sites of multiple amacrine cell synaptic inputs. **C**: Rod bipolar cell 8586 synapses onto AI AC 4943. **D**: AI AC 4943 onto synapses rod bipolar cell 5923. E: G+ AC 7188 makes a conventional synapse on AI AC 4943. **F**: AI AC 4943 receives serial conventional synapses. E denotes glutamate; G denotes glycine. The letter colors match the profiles in the image. The main panel is from section 168 and the yellow panel insert is from section 165.

In the reconstruction of AII amacrine cell fields, we encountered numerous instances of synapses between identified pairs of amacrine cells. The classic view of amacrine cells defines them as feedforward and feedback elements at bipolar cell terminals, though considerable data have long suggested otherwise. For example, the AII amacrine cell pathway receives extensive γ+ amacrine cell input at every level of the IPL, and AII amacrine cells provide output to a restricted subset of γ+ OFF amacrine cells. In past TEM studies, it has been difficult to trace amacrine cell networks, as chains of amacrine cell synapses rarely appear in a single section [14]. Viking enables tracing from any starting point. Since the synaptic targets of rod bipolar cells are GABAergic type AI and glycinergic type AII amacrine cells, an identified rod bipolar cell can be used to initiate a long-distance trace. For example, starting at a specific rod bipolar cell input (518), we traced AI amacrine cell C4943 (Figure 13A) back to its soma and then extended it into other processes (Figure 13B). While AI amacrine cells are known to form feedback networks with rod bipolar cell terminals (Figure 13C,D), it is less well appreciated that they form extensive feedforward inputs to AII cells. We found that AI amacrine cells also receive extensive G+ and γ+ amacrine cell input in the OFF layer. Some of these synapses are the largest we have ever found, with presynaptic densities exceeding 2000 nm along the AI cell dendrite (Figure 13E,F). This arm of the AI amacrine cell network thus includes the alternating glycine → GABA → glycine pattern in two separate chains bridging OFF cone and ON rod channels.

(1) OFF BC → G+ AC → AI → rod BC → AII → OFF BC

(2) OFF BC → G+ AC → AI→ AII → OFF BC

**Figure 14:**
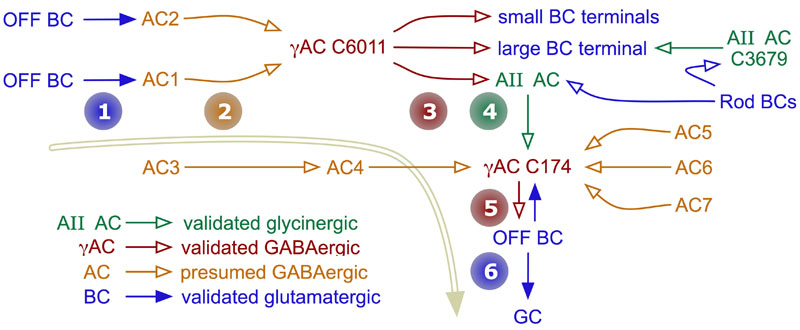
The rabbit inner plexiform layer contains synaptic chains up to six stages long. The chain starts with OFF cone bipolar cells (1) targeting two amacrine cells (2) that both converge on γ+ amacrine cell C6011. C6011 then targets two different classes of OFF cone bipolar cells and (3) a G+ AII amacrine cell, which then targets (4) γ+ amacrine cell C174, ultimately pre-synaptic (5) to another OFF cone bipolar cell that drives (6) a retinal ganglion cell.

**Figure 15:**
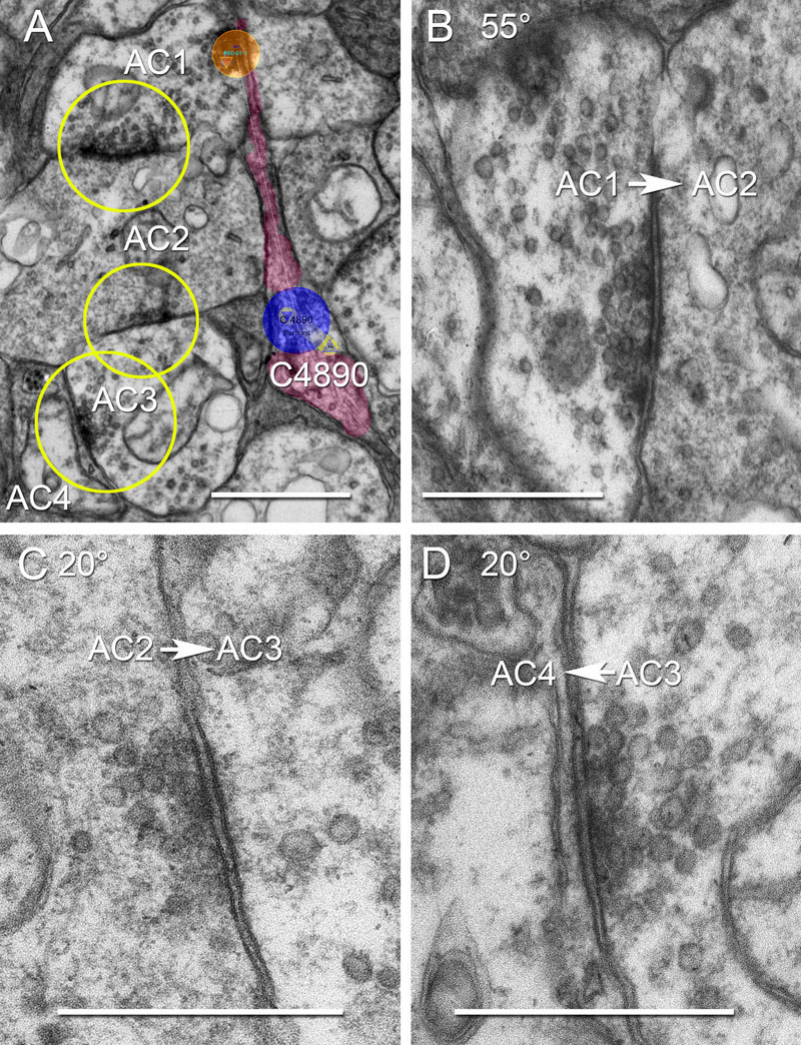
The ON starburst amacrine cell stratum of the inner plexiform layer also contains long synaptic chains. **A**: Viking screen capture at locus x 59627, y 34285, z 240 shows four amacrine cells (A1, A2, A3, A4) forming a concatenated chain (yellow circles) in the stratum occupied by the dendrites of ON starburst amacrine cell C4890 (magenta). C4890 is also postsynaptic to AC1 (orange circle) in this and other sections. The synaptic chain was re-imaged at high-resolution (0.5 nm/pixel) and goniometric tilt in panels **B**-**D**. **B**: The AC1 → AC2 synapse viewed with 55° tilt. **C**: The AC2 → AC3 synapse viewed with 20° tilt. **D**: The AC3 → AC4 synapse viewed with 20° tilt. The scale for panel **A** is 1000 nm; for panels **B**-**D** it is 500 nm.

Such complex chains are also common throughout the cone-driven strata of the IPL. For example, the OFF BC → AC (unidentified)→ γ+ AC → G+ AC → γ+ AC → OFF BC chain of Figure 14 suggests that the surrounds of bipolar cells are built from rich amacrine cell assemblies rather than simple feedback from one type of amacrine cell. Similarly, concatenated chains are abundant in layers containing ON starburst amacrine cell dendrites. In Figure 15, a four-element chain (AC1 → AC2 → AC3 → AC4) is initiated by an amacrine cell (AC1) that itself receives both bipolar cell and amacrine cell inputs (not shown) and contacts starburst amacrine cell C4890. The chain is at least six amacrine cells long, as it extends at least one more element (AC4 → AC5) many slices away in the volume and AC1 is driven by another amacrine cell. In mapping these amacrine cell chains, we have found that alternating GABA → glycine and glycine → GABA motifs are common. As shown above, γ+ neurons in the OFF sublayers are both sources and targets for AII amacrine cells. Further, γ+ AI amacrine cells heavily target G+ AII amacrine cells in the proximal rod bipolar cell layer, but are themselves targeted by another class of OFF bipolar cell-driven G+ amacrine cell in the distal IPL (Figure 13E).

### Correlating activity and structure

**Figure 16:**
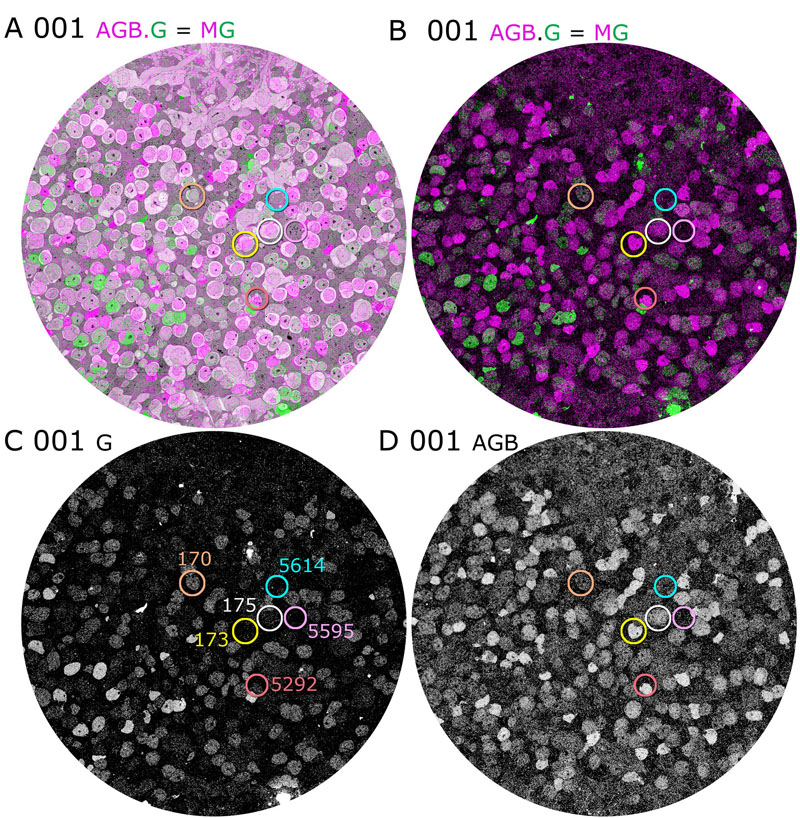
AGB mapping allows visualization of bipolar cell light responses. **A**: Slice 001 transmission electron microscope (TEM) with an overlay of glycine:AGB → magenta.green mapping shows a collection of mapped bipolar cells. **B**: Slice 001 with glycine:AGB → magenta.green mapping alone shows only the molecular signatures of the cells. **C**: Slice 001 with greyscale glycine intensity mapping reveals both glycinergic amacrine cells and bipolar cells coupled to AII amacrine cells. **D**: Slice 001 with greyscale AGB intensity mapping displays the light responses of all bipolar cells. Six identified bipolar cells are circled: one G+ WF (wide-field) bipolar cell (170), two OFF bipolar cells (173, 175), two rod bipolar cells (5595, 5614) and one non-coupled ON layer bipolar cell (5292).

**Figure 17:**
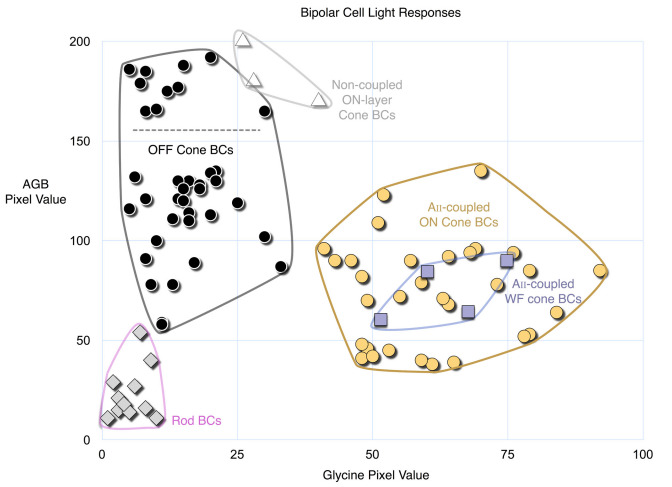
Bivariate glycine (abscissa) and photopic light-stimulated AGB signals (ordinate) for the validated bipolar cells shown in Figure 14 form unique clusters. OFF cone bipolar cells (black) have some of the strongest light-driven responses, while the mean response of most coupled ON cone bipolar cells (orange) is weaker. WF bipolar cells form a small subgroup within the entire G+ ON bipolar cell cluster (blue). Rod bipolar cells show no significant response. Conversely, three non-coupled, G- cone BCs terminating high in the ON layer have extremely strong responses (white). The stimulus regime was a 3 Hz pulse train of 3 yellow/black pulse cycles followed by one blue/black pulse cycle with a 50% duty cycle over 90 min.

Besides tracing neural networks, the combination of ATEM, CMP, and excitation mapping allows the linking of function, molecular identities, and network embedding. By activating signaling pathways in the rabbit retina with flickering blue/yellow lights and mapping them with AGB, we explored photopic response currents for each structural class of cell. A complete analysis is more extensive than can be addressed here, but we can broadly summarize our quantitative findings for the bipolar cell cohort. OFF bipolar cells are the most light-responsive and rod bipolar cells the least light-responsive neurons in this stimulus paradigm (Figure 16, Figure 17). We traced the axonal arborization and contact patterns of 95 bipolar cells in RC1 and mapped both their small molecule and AGB signatures. G+ bipolar cells all arborize in the known ON cone bipolar cell stratum of the IPL and all make observable gap junctions with AII amacrine cells (e.g., Figure 5, Figure 6). The robustness of this observation is extended by our ability to quantify glycine signals in every bipolar cell (Figure 16). Of the bipolar cells that terminate in the nominal ON sublayer, those with gap junctions onto AII amacrine cells are all G+. The raw, unprocessed 8-bit mean pixel values (PVs)±1SD for the glycine channel differentiates coupled and uncoupled bipolar cells: ON bipolar cells with gap junctions, PV 63±13 (n=32); OFF bipolar cells with no gap junctions, PV 15±7 (n=42); and rod BCs, PV 5±3 (n=12). Rare instances of apparently uncoupled, presumed ON bipolar cells (based on the level of IPL branching and high AGB response, not shown) had low but significant glycine levels, PV 31±7 (n=3). Signal-to-noise ratios from normal distribution overlap show that the probability of misclassifying an ON cone bipolar cell as an OFF cell based on glycine signals is less than 0.015; the probability of misclassifying an OFF bipolar cell as a G+ ON bipolar cell is less than 0.02. The difference in AGB signals is even greater. Each major class of bipolar cells likely expresses different glutamate receptors or ionic selectivities, and they are strongly distinguished by their AGB responses to pan-spectral photopic flicker. We define five superclasses of rabbit retinal bipolar cells based on level of termination, contact patterns, and small molecule signature: (1) G- OFF bipolar cells lacking heterocellular gap junctions with AII cells and terminating in the distal IPL (axons 1–10 μm long); (2) G+ ON cone bipolar cells displaying heterocellular gap junctions with AII cells, terminating in the proximal IPL (axons 9–17 μm long); (3) G+ wide-field bipolar cells with axonal arbors just distal to the rod bipolar cell terminals displaying both heterocellular gap junctions with and ribbon synapses onto AII cells; (4) G- rod bipolar cells directly driving AII amacrine cells terminating deep in the proximal IPL (axons 12–17 μm long); and (5) a few G- bipolar cells of the upper half of the ON layer lacking any association with AII cells. All of these superclasses are statistically distinguished by AGB signals (Figure 15, Figure 16). OFF cone bipolar cells have strong light-driven signals, suggesting that their AMPA or kanic acid (KA) receptor cohorts have large unitary conductances or that the receptor number is high. G+ ON cone bipolar cells have moderate AGB signals, roughly half the pixel value of the OFF bipolar cell cohort. Given that the cells experienced the same stimulus regime, this implies that currents ultimately gated by mGluR6 receptors of coupled ON cone bipolar cells are significantly smaller and, using the scaling previously established for AGB immunodetection [3], corresponds to a 2.8-fold difference in AGB current. Paradoxically, the small sample of noncoupled ON cone bipolar cells exhibit the highest signal strengths of all. AGB rod bipolar cells have almost no detectable AGB signal and wide-field bipolar cells have distinct but very weak signals.

### Building connectomes

**Figure 18:**
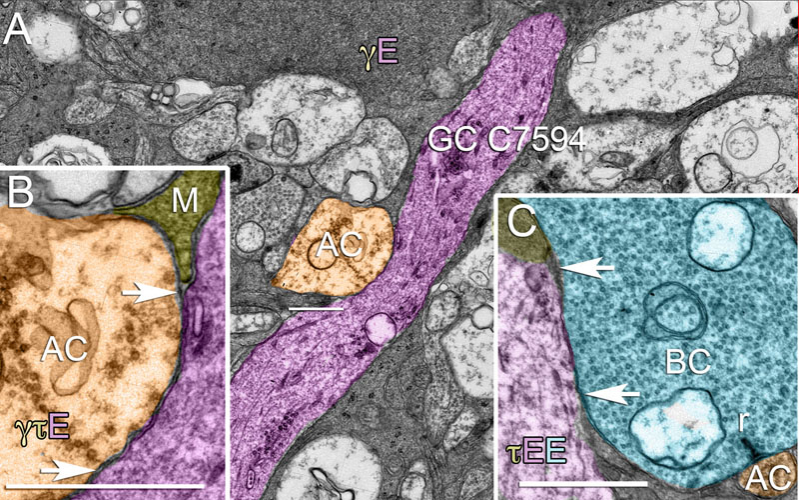
Neuronal processes are often apposed without synaptic contacts. **A**: A dendrite (purple) from ON ganglion cell C7594 courses through the inner plexiform layer and is physically apposed to many cells with which it never makes synaptic contact, such as an amacrine cell dendrite (AC, orange). **B**: An enlarged view of the apposition shown in panel **A**. The amacrine cell membrane is directly apposed to ganglion cell C7594 with no intervening glial processes (M). The image is centered on x 71300, y 46622, z 279 in RC1, the apposition spans sections 275–286 (770–990 nm), and is at least 1450 nm long in the XY plane. **C**: An apposition between an ON cone bipolar cell (BC, azure) and ganglion cell C7594 at a more distal location in RC1. E denotes glutamate; γ denotes GABA; τ denotes taurine. The letter colors match the profiles in the image. The bipolar cell makes a ribbon monad onto a very small amacrine cell dendrite, but is never presynaptic to the ganglion cell. The image is centered on x 73722, y 53700, z 258 in RC1, spans sections 256–269 (910–1170 nm), and is at least 2200 nm long in the XY plane based on serial tracking. Scales, 1000 nm.

After over 300,000 annotations, tagging over 12,000 individual structures (cells, synapses, gap junctions), rendering over 100 cells, reassessing of the AII amacrine cell network, and viewing over 350,000 individual TEM images in multiple contexts, several factors emerged that impact the theory and practice of building accurate network diagrams from ATEM data. The first is that synaptic resolution is essential. Without it, neither the scope of the axonal synapse networks nor the extreme complexity of amacrine cell chains would have been revealed. Until the dense connections classified as gap junctions were validated by goniometric high-resolution viewing, assertions about their presence (especially unexpected presence) or absence in a network would have been dubious. Many processes would have been impossible to trace without 2 nm lateral resolution. However, adjacency and connectivity are not equivalent. We had assumed that adjacency would imply ultimate connectivity at some point, so that lower resolution optical or scanning electron microscope imaging might be adequate for network characterization. Our connectome data show that this is untrue for several reasons. Without 2 nm resolution, it is impossible to visualize fine glial processes that may be interposed between candidate neural processes. More importantly, fictive adjacency is the norm in the retina: direct membrane appositions occur between neighboring processes that never make any synaptic contacts. These are far more common than synaptic contacts. Figure 18 shows examples from the extensively traced ON ganglion cell C7594. As it passes laterally through the IPL, ganglion cell C7594 encounters numerous amacrine (e.g., Figure 18A,B) and bipolar cell profiles (e.g., Figure 18C) that make synapses onto nearby targets, but make no synapses onto C7594, despite long stretches of uninterrupted contact in 3D space. Pairs of neurites often approach each other and Müller cell sheets pull back to expose both neuronal membranes, though no specializations of any kind (synapses, membrane densities, cisterns, gap junctions, etc.) ever occur. This cannot be proven unequivocally in a single slice, but is clear in 3D reconstructions. Further, cells actively forming synapses onto one target will bypass another altogether and never contact it. Many ganglion cells directly appose rod bipolar cell synaptic terminals, with no interposed glia, but rod bipolar cells never make synapses onto them. On balance, synaptic contacts are rare among adjacent processes and must be mapped by visualization, not geometric inference.

## Discussion

### AII amacrine cells

To validate our approach for building connectomes, we revisited the specific connectivities of AII amacrine cells, largely affirming previous findings in terms of rod bipolar cell inputs, OFF cone bipolar cells, and coupling to ON cone bipolar cells. We were able, however, to extend previous findings. First, we found that AII cells had additional photopic ribbon inputs from wide-field ON cone bipolar cells. Thus, all superclasses of bipolar cells drive AII cells via ribbon synapses: rod, wide-field ON cone, and several classes of OFF bipolar cells. The wide-field ON cone bipolar cell inputs to AII cells were likely missed by earlier workers because overlapping rod and wide-field ON cone bipolar cell terminals are poorly distinguishable without reconstruction and in the presence of high-contrast ferrocyanide intensification. The presence of such inputs partially explains why light-adapted AII cells remain ON-center in polarity [31], even when driven by high-gain OFF cone bipolar cell ribbon synapses [15,22] compared to low-gain, attenuated gap junctions from bipolar cells [32]. Pang et al. [33] have recently shown that some mouse rod bipolar cells have direct physiologic cone inputs, which would further strengthen AII cell ON polarity if rabbit rod bipolar cells showed the same bias. This is possible, since Dacheux and Raviola showed by Golgi-TEM [34] that rabbit rod bipolar cells occasionally contact cones. On balance, the direct photopic ON bipolar cell synaptic drive to AII cells may be substantial. Wide-field ON bipolar cells are also G+ and make gap junctions with AII cells. Famiglietti argues that they are blue-cone selective [35]. While we cannot affirm this in volume RC1, it is consistent with the observations of Field et al. that primate blue-sensitive small bistratified ganglion cells also show substantial rod responses [36], which could arise via gap junctions between AII cells and blue-cone selective bipolar cells. However, reconstructions of primate blue-cone bipolar cells make no mention of gap junctions [37], and multiparametric clustering of cone bipolar cells in the cat IPL reveals cells that do not couple to AII cells [38]. Sorting out the blue-cone bipolar cells, wide-field cells, and coupling patterns will require direct knowledge of identified cone contacts. However, for the time being, the presence of direct synaptic input from ON cone bipolar cells to AII cells is clear.

AII cells receive extensive amacrine cell synapses at every level of the IPL [15], and CMP shows that this drive arises from γ+ amacrine cells. AII cell lobular appendages in the cone OFF sublayer and arboreal dendrites in the cone ON sublayer were postsynaptic to different amacrine cells, suggesting that AII cells are more than scotopic fanout devices, and perhaps provide general ON → OFF photopic crossover network functions shared by most vertebrates (see below). As many as five distinct classes of amacrine cells provide inputs to AII cells. Paradoxically, AII cells only show strong, large surrounds when dark adapted [39]. This might be expected, as the low-pass surround pathways of light-adapted mammalian ganglion cells do not appear to be GABAergic [40], even though they receive extensive GABAergic amacrine cell input. The abundant amacrine cell synapses of cone pathways likely function in a higher spatiotemporal frequency domain. Indeed, spatial noise analysis and direct current injection studies in catfish show that amacrine → ganglion cell signaling is fast, complex, and spatially constrained [41,42].

AII cells also receive inputs from processes rich in dense-core vesicles characteristic of peptidergic neurons in the distal IPL. Candidate peptides include neuropeptide Y, vasointestinal peptide, and somatostatin [43]. These neurons have two kinds of contact sites: one kind where dense core vesicles appear to be fusing, and another where conventional small, clear vesicles form classical presynaptic appositions (Figure 5C,D). AII cells also receive axosomatic synapses, presumably from tyrosine hydroxylase immunopositive (TH1) cells [28]. While it is thought that dopamine modulates AII amacrine cells [32], the axosomatic synapses of AII amacrine cells (Figure 5B) are structurally characteristic of fast transmitter systems and are likely glutamatergic in the brain [44] and retina [29], contrary to previous hypotheses. TH1 cells in the rabbit retina are γ- and glutamate rich, and the axosomatic synapses on AII cells are γ- (Figure 7, Figure 8). In a full N-dimensional classification space, TH1 cell small molecule signatures are indistinguishable from those of retinal ganglion cells, and incompatible with those of any other known amacrine cell (not shown).

While most of the signaling from AII amacrine cells to OFF layer ganglion cells flows through AII → OFF bipolar → ganglion cell chains, direct AII amacrine → OFF ganglion cell synapses are present, consistent with the physiologic findings of Beaudoin et al. [45]. The low percentage of AII amacrine cell synapses onto ganglion cells reported by earlier researchers [15] is somewhat misleading, as bipolar cell terminals are simply numerically dominant. However, certain OFF GC dendrites (e.g., cell 5150) collect numerous, large AII amacrine cell synapses with postsynaptic densities ranging from 500 to 900 nm in diameter. Only a subset of OFF ganglion cells are targets of AII amacrine cells. The details of this connectivity will be the subject of future papers. Similarly, the suggestion that AII ACs do not contact amacrine cells [15] is also incorrect. Most amacrine cells in the OFF layer do not receive any input from AII cells, but a subset of GABAergic amacrine cells is selectively targeted by them. That too will be the subject of future papers.

Finally, as the arboreal dendrites of AII amacrine cells traverse and terminate within the rod bipolar cell-driven scotopic ON layer, they are postsynaptic to rod bipolar cells, receive extensive inputs from γ+ AI rod amacrine cell and γ+ ON cone ACs, and form large AII-AII homocellular gap junctions (Figure 9). There is a significant difference between the homocellular gap junctions and heterocellular gap junctions of AII cells, noted by Strettoi et al. [15]. Conversely, homocellular gap junctions show little evidence of scaffolding protein accumulation, while heterocellular gap junctions with bipolar cells show significant scaffolding protein density, with thicker layers on the AII face. In total, considering dopaminergic and conventional inputs from TH1 cells, and peptidergic and conventional inputs from peptide/γ+ amacrine cells to be distinct channels, AII amacrine cells make no fewer than 17 different kinds of cellular associations in the IPL (Figure 6). The presence of asymmetric cytoplasmic densities at the AII-ON bipolar cell gap junctions implies a connexin-associated protein aggregation that does not occur at AII-AII gap junctions. Han and Massey [46] showed that most cone ON bipolar cells in the mouse retina lack Cx36 expression, while AII amacrine cells clearly use Cx36 for AII-ON bipolar cell coupling. This and other evidence [32] suggest that most AII-ON bipolar cell instances likely involve heterotypic connexin pairings. While this makes sense in terms of the observed asymmetric densities, it is not a mechanistic explanation. The fundamental question remains: How do AII amacrine cells selectively associate scaffolding and adaptor proteins at heterocellular plaques involving Cx36 but not at homocellular Cx36 plaques? While connexin plaques are known to associate with a range of proteins such as tight junction elements (e.g., ZO-1), various kinases, signaling intermediates (e.g., β-catenin), and internalization proteins (e.g., caveolin), the signals that facilitate aggregation are poorly known [47]. The differential density implies transjunction signaling through associated cadherin or other adhesion protein signaling in parallel, direct connexin-connexin binding, associated cadherin or other adhesion protein signaling in parallel, or via some bipolar cell-specific small molecule signal. In any case, the observation supports the notion that AII-ON bipolar cell gap junctions are regulated differently from AII-AII junctions [32].

### Axonal ribbon synapses and “veto” synapses

The axons of cone bipolar cells form distinct zones of synaptic integration. We show that >90% of axonal nanoribbons target amacrine cells, although we also observed noncanonical ON cone bipolar cell axonal inputs in the OFF sublayer onto bistratified ganglion cells that also receive conventional bipolar cell synapses in the ON layer (not shown). Using optical techniques, similar noncanonical OFF layer contacts have recently been described targeting melanopsin ganglion cells and TH1 cells and appear functional [30,48]. The abundance of axonal synapses (Figure 12) suggests a major role in shaping receptive fields. Furthermore, multistratified ribbon outputs from cone bipolar cells have long been known in nonmammalians [49].

Cone bipolar cells also display abundant axonal amacrine cell inputs that we provisionally term axonal “veto” synapses. We define candidate veto synapses as those uniquely in a spatial position to substantially modify signal flow in a cell. In the case of bipolar cells, we consider large GABAergic synapses on the axon proper, often closer to the soma than the axon terminal, to be candidate veto synapses. Architecturally, they are very similar to the topology of GABAergic chandelier cells, which target the axons of cortical pyramidal neurons [50]. As shown in Figure 11E, veto synapses can be as wide as the bipolar cell axon (>1 μm) and the total postsynaptic area can exceed 1 μm^2^. This is a substantial postsynaptic size compared to most amacrine cell synapses onto bipolar cell axon terminals (e.g., Figure 13D), where the typical area is less than 0.2 μm^2^. Such synapses, singly or in clusters, could modify bipolar cell potentials by global hyperpolarization or local shunting. Their function remains unknown, but the existence of both axonal ribbon and veto synapses suggests that electrotonic lengths of complex, highly branched mammalian cone bipolar cells might be neither large nor constant, as presumed from earlier sharp electrode recordings of fish bipolar cells [51]. Given the large synaptic surfaces of the cone bipolar cell terminals, they likely have a lower impedance than their long, thin axons. Considering these topologies as dendritic spines [52] raises the possibility that modulating axon impedance (analogous to the spine shaft) could gate signal transmission from bipolar cell soma → terminal or vice versa. Viewed as single instances in a TEM image, these axonal ribbon and veto synapses would be unremarkable, as there would be no way to know that they were associated with bipolar cell axons instead of other processes such as ramifying bipolar cell terminals. Connectomics makes the case for their existence unambiguous.

### Network complexity

The functions of the serial amacrine cell synapses described by Dowling [53] have never been satisfactorily resolved. Nested and concatenated motifs found abundantly in teleost retinas [14] have not been similarly explored in mammals. RC1 clearly displays extensive amacrine cell concatenation. Glycine → GABA and GABA → glycine motifs are common (Figure 4, Figure 6, Figure 13), consistent with recent physiologic data for alternating pharmacologic drive [54-57]. In particular, every G+ amacrine cell we have found in RC1 is both presynaptic and postsynaptic to γ+ amacrine cells. This suggests that the surrounds of most or all ganglion cells are built from pharmacologically complex chains instead of simple BC → γ+ AC → BC networks. As shown above, AII cells both receive from and target γ+ amacrine cells. Furthermore, AI cells that target AII cells in the rod bipolar cell layer of the IPL are themselves driven by G+ OFF amacrine cells (Figure 13), creating a chain of OFF G+ AC → ON γ+ AI AC → ON G+ AII AC → γ+ GABA AC signaling, mixing scotopic/photopic and GABAergic/glycinergic signals.

The surround organizations of both ON and OFF bipolar ganglion cells clearly engage numerous long amacrine cell chains (Figure 14, Figure 15), though their functions remain unknown. There are four basic synaptic motifs that could lead to long chains: (1) Reentrant signaling (Aj ⇄ Aj), where an amacrine cell targets its own class and implicitly generates long chains. Encountering long chains in a very local region suggests that both the coverage and the Hausdorff dimension [58] of the dendritic arbor for Aj are high. (2) Reciprocal amacrine cell signaling (Aj ⇄ Ak) is rare, but we have found it involving an interstitial ON-OFF γ+ AC in RC1 and it has been previously reported in goldfish retina for glycinergic amacrine cells [59]. It too will generate infinitely long chains depending on the dual coverages and Hausdorff dimensions of Aj and Ak. (3) Looping signaling (Aj → Ak → Am → Aj) gives each cell a nonreciprocal, nonreentrant target but potentially creates very long chains. This specific motif has not yet been found. (4) Finally, simple chains of n distinct classes (Aj → … → An) will create chains of exactly n steps. The ON layer associated with starburst amacrine cells is replete with very long chains (Figure 15), with at least six steps in a local region. We think it is unlikely that a simple chain will involve six distinct cell classes. Type 1 reentrant signaling via direct starburst → starburst connections [60] explains the topology, consistent with the evidence of direct inhibitory GABA synapses between starburst cells as shown by Lee and Zhou [61].

Another feature of network complexity is crossover, a mechanism proposed by Werblin and colleagues for sign-inverting signal transfer between ON and OFF channels to correct for synaptic rectification [62,63]. Narrow field glycinergic amacrine cells such as AII cells are some of the most likely candidates to mediate crossover, since they likely have significant photopic ON pathway drive and synaptic outputs to many types of OFF pathway components, including most OFF bipolar cells, several OFF amacrine cells and some kinds of OFF ganglion cells.

### Molecular markers and activity

RC1 contains molecular markers that have previously been shown to be useful in categorizing retinal cells [8] at the optical level. By using multichannel classification based on GABA, glycine, glutamate, taurine, and glutamine signals, every cell can be grouped into one of several major classes of retinal neurons. The inclusion of the excitation mapping marker AGB in vivo with optical stimulation (alternating yellow and blue flashes in this case) embeds an additional signal that allows further segmentation. We here demonstrate that G+ bipolar cells correspond identically to ultrastructurally identified ON cone bipolar cells that make gap junctions with AII amacrine cells. In addition, different bipolar cell classes show vastly different light-driven AGB responses, suggesting that each bipolar cell is tuned for various stimulus conditions and provides corresponding output drive. AGB is a channel permeant cation with high selectivity for glutamate-activated AMPA, KA, and N-methyl-D-aspartate (NMDA) receptors, as well as unactivated mGluR6-gated channels; its signal strength represents the time integral of the glutamate input drive [8-12]. Under the light stimuli used here, OFF bipolar cells showed the largest AGB signals and, by extension, the largest light-activated currents, similar to our findings using AMPA and KA drive [11]. One intriguing outcome was the virtual absence of AGB signals in rod bipolar cells. Since this bright photopic stimulus should have saturated rod photoreceptors, the glutamate release should have been extremely low and, by extension, the mGluR6-gated cation conductance and AGB permeation should have been maximal in rod bipolar cells. This suggests that either saturated rod photoreceptor glutamate release was paradoxically high or rod bipolar cells have an adaptive mechanism to shut down their mGluR6-gated cation conductances in the photopic state. The former is not likely the case, as high rod glutamate release would also have generated high AGB signals in horizontal cell axon terminals by activating AMPA receptors. The horizontal cell axon terminals had negligible AGB signals (not shown). In contrast, G+ ON cone bipolar cells showed moderate to strong AGB signals, on average less than uncoupled cells. We also detected three G- ON cone bipolar cells, which had the largest AGB responses of all cells in RC1. These may correspond to the physiologically [64] and immunocytochemically [65] uncoupled bipolar cells previously described in mammals. If these cells also display only mGluR6-gated signaling, it suggests that part of the low signal currents in G+ bipolar cells arises from either direct loss of AGB into coupled cells by diffusion and/or that the lower impedance of coupled cells reduces the driving force for AGB current through channels.

These results suggest a next stage of analysis that we have only begun: response correlations across connected neurons. For example, OFF bipolar cell 325 has a high AGB signal, is a finely branched OFF bipolar cell with few ribbons, and is connected to AII amacrine cell 6153 and γ+ amacrine cell 115. However, cells 6153 and 115 both have moderate AGB signals (data not shown). These amacrine cells also receive ribbon synapses from other classes of OFF cone bipolar cells and, thus, their response properties are likely represent a combination of bipolar cell input strengths and their own iGluR profiles. It is our hope that we can eventually build a full matrix of response correlations once the connectivity mapping of RC1 is complete.

### New perspectives on networks

Network graph theory provides a new context for analyzing retinal networks. Connectomics provides a tool to map connections into graphs and discover new communication modes. For example, the connections we refer to as cistern contacts were discovered long ago [66], but their functions remain unknown. However, we can now map their relationships across neurons and perhaps develop strategies for screening them with molecular markers. The richness of retinal networks (synapses, gap junctions, and possibly cistern contacts), coexisting fields of sparse and dense synaptic contact motifs, high connection specificities [e.g., 67], a high rate of unrealized synaptic opportunities (Figure 18), and the requirement that retinal topology conform to imaging sampling rules forces the retina to be a sparse multigraph with many vertices (cell contact points) of very low connection degree [68], similar to very large scale integrated (VSLI) circuits. A low connection degree means that retinal networks display many fewer connections at certain vertices than theoretically possible [69]. This sparseness may shed some light on our expectations of connection statistics within cell groups. For example, why are axonal ribbons not present in all ON cone bipolar cells? While one obvious answer would be that there are different kinds of ON cone bipolar cells with varied connection rules, it is also likely that not all axons intersect every correct candidate target. Importantly, there are no null ribbons, i.e., membrane-associated ribbons without postsynaptic partners. Thus, the driving mechanism is more likely geometry rather than simple connectivity. Long, straight neurites of radiate amacrine cells in fish [70] and certain wide-field amacrine cells in mammals [71] (e.g., AI cells, S1 type) cannot geometrically contact all members of a target class without strongly curving. Thus, cell coverage determines the number of vertices successfully hit by the connecting edges, not the number of edges passed. Why should we care about sparse multigraphs? If sparse connectivity underlies the architecture of retina and brain organization, the precision of contacts needs to be very high [72]. There are two polar concepts for wiring systems: statistical and precise. Statistical networks reach consensus by averaging signals and allowing errors, sometimes at high rates. Precise networks are just that: networks with few errors and strong connection rules. As we explore RC1, we will be able to determine the degree and the nature of variation across individual copies of a single cell class (e.g., AII amacrine cells) or within a network.
